# Resilience and vulnerability of maternity services in Zimbabwe: a comparative analysis of the effect of Covid-19 and lockdown control measures on maternal and perinatal outcomes, a single-centre cross-sectional study at Mpilo Central Hospital

**DOI:** 10.1186/s12884-021-03884-5

**Published:** 2021-06-04

**Authors:** Clare Shakespeare, Handsome Dube, Sikhangezile Moyo, Solwayo Ngwenya

**Affiliations:** 1 Department of Obstetrics and Gynaecology, Mpilo Central Hospital, Vera Road, Mzilikazi, Bulawayo, Zimbabwe; 2 Maternity Department Matron, Mpilo Central Hospital, Vera Road, Mzilikazi, Bulawayo, Zimbabwe; 3 National University of Science and Technology, Bulawayo, Zimbabwe

**Keywords:** Covid-19, Pandemic, Indirect maternal outcomes, Indirect perinatal outcomes, Vulnerability, Resilience, Healthcare systems

## Abstract

**Background:**

On the 27^th^ of March 2020 the Zimbabwean government declared the Covid-19 pandemic a ‘national disaster’. Travel restrictions and emergency regulations have had significant impacts on maternity services, including resource stock-outs, and closure of antenatal clinics during the lockdown period. Estimates of the indirect impact of Covid-19 on maternal and perinatal mortality was expected it to be considerable, but little data was yet available.

This study aimed to examine the impact of Covid-19 and lockdown control measures on non-Covid outcomes in a government tertiary level maternity unit in Bulawayo, Zimbabwe, by comparing maternal and perinatal morbidity and mortality before, and after the lockdown was implemented.

**Methods:**

This was a retrospective, observational study, using a cross-sectional design to compare routine monthly maternal and perinatal statistics three months before and after Covid-19 emergency measures were implemented at Mpilo Central Hospital.

**Results:**

Between January-March and April-June 2020, the mean monthly deliveries reduced from 747.3 (SD ± 61.3) in the first quarter of 2020 to 681.0 (SD ± 17.6) during lockdown, but this was not statistically significant, *p* = 0.20. The Caesarean section rates fell from a mean of 29.8% (SD ± 1.7) versus 28.0% (SD ± 1.7), which was also not statistically significant, *p* = 0.18. During lockdown, the percentage of women delivering at Mpilo Central Hospital who were booked at the hospital fell from a mean of 41.6% (SD ± 1.1) to 35.8% (SD ± 4.3) which was statistically significant, *p* = 0.03. There was no significant change, however, in maternal mortality or severe maternal morbidity (such as post-partum haemorrhage (PPH), uterine rupture, and severe preeclampsia/eclampsia), stillbirth rate or special care baby unit admission. There was an increase in the mean total number of early neonatal deaths (ENND) (mean 18.7 (SD ± 2.9) versus 24.0 (SD ± 4.6), but this was not statistically significant, *p* = 0.32.

**Conclusions:**

Overall, maternity services at Mpilo showed resilience during the lockdown period, with no significant change in maternal and perinatal adverse outcomes, with the same number of man-hours worked before and during the lockdown Maternal and perinatal outcomes should continue to be monitored to assess the impact of Covid-19 and the lockdown measures as the pandemic in Zimbabwe unfolds. Further studies would be beneficial to explore women’s experiences and understand how bookings and deliveries at local clinics changed during this time.

## Background

In March 2020, Covid-19 was recognised by the World Health Organisation (WHO) as a global pandemic. Although Africa appears so far to have been comparatively spared [1], limited health system capacity for testing and disease surveillance restricts assessment of the true size and impact of the pandemic in many African countries [2]. Widespread use of non-pharmacological measures to control the pandemic include international, regional and local travel restrictions, school and workplace closures, and limitations on social gatherings. Whilst likely cost-effective in high income countries [3], the feasibility and effectiveness of such lockdown interventions has been questioned in the context of sub-Saharan Africa with younger population age structures, predominance of informal economies and weak existing healthcare systems [4, 5].

Despite low numbers of cases, and limited direct morbidity and mortality related to Covid-19, health care systems in many southern African countries remain vulnerable to indirect effects on non-Covid-19 health outcomes, especially maternal and new born health [6]. Fear of the virus, shortages of resources, and disruption of healthcare infrastructure and systems as a result of lockdown measures, present increased barriers for access to healthcare services and may result in breakdown of routine health programmes [6, 7]. Similar issues during the Ebola outbreak in West Africa in 2014 resulted in a sustained reduction in use of essential maternal and child health services [8]. In some cases, indirect effects on health were more extensive than the epidemic itself [9]. Already in some European countries reduction in use of child health services has been observed during the Covid-19 pandemic, raising fears about additional morbidity and mortality resulting from delayed presentations [10, 11]. Models based on estimates of disruption to routine maternal and child health services and food insecurity as an indirect result of Covid-19 in low and middle income countries (LMICs) predict up to 38.6% increase in maternal deaths per month across 118 countries [6].

Zimbabwe has had a limited pandemic so far, with 1478 cases and 25 deaths as of 19^th^ July 2020 [12]. Fewer than ten cases have been officially reported in pregnant women (Mpilo Rapid Response Team, personal communication, 20^th^ July 2020). The Zimbabwean government declared Covid-19 a national disaster on the 27^th^ March 2020. A nationwide lockdown commenced on 1^st^ April. All non-essential travel, shops and services were suspended for 5 weeks, with gradual relaxation of measures since to re-open shops and businesses. Although healthcare was always exempted from lockdown measures, ongoing issues for healthcare services included transport difficulties for patients and staff, and financial hardship resulting from loss of livelihoods. These difficulties presented additional barriers for affordability and access to healthcare.

Mpilo Central Hospital is a government tertiary referral hospital in Bulawayo. The maternity department receives high risk cases and referrals from the whole of southern Zimbabwe (Matabeleland North, Matabeleland South and Midland provinces). It is a 1000-bedded hospital, and its maternity unit delivers 8000–10 000 babies per year. The maternity unit is a 257-bedded, consultant-led unit with 235 midwives and 30 medical staff working in the unit. Many essential services, including antenatal clinics, had closed and all elective work, including elective Caesarean sections, were cancelled during the lockdown due to the Covd-19 pandemic.

An increasing number of individual studies and systematic reviews are providing evidence on the impact of Covid-19 on pregnancy outcomes [13, 14]. Little is available on the indirect impacts on maternal and perinatal morbidity and mortality, especially in sub-Saharan Africa. This study aimed to examine the impact of Covid-19 and the lockdown control measures on non-Covid outcomes at Mpilo Central Hospital in Bulawayo, Zimbabwe. The objectives were to compare maternal and perinatal outcomes before and after lockdown was implemented. The hypothesis was that both morbidity and mortality would have increased.

## Materials and methods

This was a retrospective, observational study, using a cross-sectional design to compare routine monthly maternal and perinatal statistics before and after the implementation of Covid-19 lockdown measures at Mpilo Central Hospital in Bulawayo, Zimbabwe.

Maternal and perinatal statistics are routinely collected every month from hospital records and daily reports. Records are kept as paper-based registries in clinical areas, with data entered by ward staff. Monthly totals are recorded by ward in-charges, and checked by reproductive health officers who collate and present the results. Staff attendance registers were used to calculate man-hours. These routine data were used to compare outcomes for three months before and after the lockdown (January-March vs. April-June 2020) was implemented. These months were chosen as a comparator to keep other factors as similar as possible as Zimbabwe has experienced many other disruptions to services which may affect outcomes, such as industrial action by medical staff during 2019. All women that delivered during the study period were included in the study. Data were double-checked against original registry entries. Before analysis the data were cleaned, coded and entered into a Microsoft Excel spreadsheet, then exported to SPSS Version 20 (IBM, Armonk, NY, USA) for analysis as appropriate. Descriptive statistics were performed and presented as frequencies and percentages for categorical variables. The Mann–Whitney U test was used for comparing the means for the man-hours. A two-sample z-test for difference between proportions was used for statistical analysis where appropriate, comparing three-month average outcome values. A *p* value of < 0.05 was considered to be statistically significant.

Variables were chosen to reflect the following aspects of maternity care; workload, antenatal care, maternal morbidity and mortality, perinatal morbidity and mortality (Table 1). According to Donabedian’s framework for assessment of quality of care [15], the selected indicators of workload and antenatal care represent process measures, while maternal and perinatal morbidity and mortality indicators represent outcome measures.

**Table 1:** Maternal and perinatal outcome indicators

Outcome	Measure	Indicator (per month)
Workload	Staff attendance	Man-hours available
	Deliveries on labour ward	Total deliveries
	Caesarean section rate	Total number of Caesarean sections
		Percentage of deliveries by Caesarean section
Antenatal care	Booking status of women delivering on labour ward	Percentage of deliveries with pregnancy booked at Mpilo
		Percentage of deliveries referred from other clinic/hospital
		Percentage of deliveries with unbooked pregnancy
Maternal morbidity and mortality	Maternal mortality	Total number of maternal deaths
	Severe maternal morbidity	Percentage of deliveries complicated by post-partum haemorrhage), severe pre-eclampsia or eclampsia, or uterine rupture
Perinatal morbidity and mortality	Stillbirth	Stillbirth rate (per 1000 deliveries)
	Neonatal morbidity	Total number of early neonatal deaths
		Special care baby unit (SCBU) admission rate

There is no global consensus on which maternal and perinatal indicators should be chosen to represent quality and outcomes of maternity care [16], these indicators were chosen to reflect and correspond with a number of reviews on the topic [16–18], as well as to fit data that is routinely recorded and available at Mpilo.

## Results

The results are presented in Table 2 and 3 below.

**Table 2:** Staff attendance showing man-hours

Months 2020	Staff attendance		Total	40 h week/man-hour	*P*-value
	Nursing	Medical			
Pre-lockdown period					
January	213	25	238	9520	
February	211	27	238	9520	
March	211	26	238	9520	
Mean				9520.0 (SD ± 0.0)	
During/post lockdown period					0.83
April	210	29	239	9560	
May	210	28	238	9520	
June	209	26	235	9400	
Mean				9506.7 (SD ± 92.4)

**Table 3:** Maternal and perinatal outcomes, Jan-March versus April-June 2020

Year 2020	Jan	Feb	March	Mean	SD	April	May	June	Mean	SD	*P*-value
**Workload**											
Total deliveries	812	690	740	747.3	61.3	688	694	661	681.0	17.6	0.20
No. of Caesarean sections	237	219	211	222.3	13.3	177	178	189	181.3	6.7	0.07
Caesarean section rate (%)	29.2	31.7	28.5	29.8	1.7	29.8	25.7	28.6	28.0	2.1	0.18
**ANC**											
Pregnancy booked at Mpilo (%)	40.6	42.8	41.5	41.6	1.1	40.8	32.9	33.9	35.8	4.3	0.03
Referred (%)	55.4	54.1	54.3	54.6	0.7	54.4	57.6	57.0	55.1	1.6	0.52
Unbooked (%)	4.6	3.8	4.9	4.4	0.6	5.1	9.9	8.9	8.0	2.5	0.01
**Maternal morbidity/mortality**											
Total number of maternal deaths	2	3	0	1.7	1.5	2	0	1	1	1.0	
Postpartum haemorrhage (%)	1.5	1.9	1.2	1.5	0.4	1.6	1.2	1.1	1.3	0.3	0.75
Severe preeclampsia/eclampsia (%)	0.9	1.7	1.5	1.3	0.4	1.6	1.4	0.6	1.2	0.5	0.74
Uterine rupture (%)	0	0	0	0	0.0	0.1	0.3	0	0.1	0.2	0.39
**Perinatal morbidity and mortality**											
Stillbirth rate/1000 live births	28.3	33.3	37.8	33.1	4.8	32.0	25.9	34.8	30.9	4.6	0.81
Total number of early neonatal deaths	17	22	17	18.7	2.9	29	20	23	24.0	4.6	0.32
SCBU admissions (%)	25.5	25.9	20.4	23.9	3.1	22.5	27.1	18.6	22.7	4.3	0.88

*Key*:* ANC* Antenatal clinic, *SCBU* Special care baby unit, *(%)* percentage, *SD* standard deviation

### Workload

There was no statistical difference in the number of man-hours worked before and after the lockdown measures were implemented (means 9520.0 (SD ± 0.0) vs. 9506.7 (SD ± 92.4), *p* = 0.83).

**Fig. 1:**
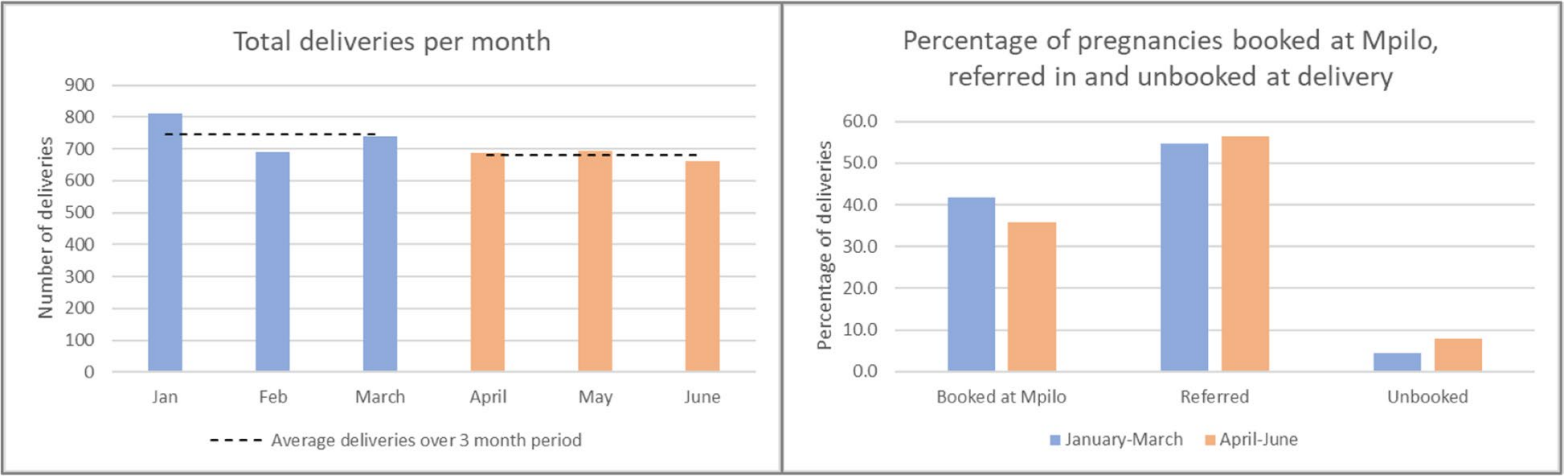
Trends in total deliveries and percentage of Mpilo booked pregnancies, referrals, and unbooked pregnancies at delivery

The mean number of deliveries conducted each month reduced from 747.3 (SD ± 61.3) in the first quarter of 2020 to 681.0 (SD ± 17.6) during lockdown, but this was not statistically significant, *p* = 0.20 (see also Fig. 1). The number of Caesarean sections also reduced, both as absolute numbers performed (mean 222.3 (SD ± 13.3) per month versus 181.3 (SD ± 6.7), but this was not statistically significant, *p* = 0.07, and as was the Caesarean section rate (29.8% (SD ± 1.7) versus 28.0% (SD ± 1.7), which was also not statistically significant, *p* = 0.18.

### Antenatal care

During lockdown, the percentage of women delivering at Mpilo who were booked at the hospital fell from a mean of 41.6% (SD ± 1.1) to 35.8% (SD ± 4.3) which was statistically significant, *p* = 0.03. The number of women presenting in labour with unbooked pregnancies almost doubled from a mean of 4.4% (SD ± 0.6) to 8.0% (SD ± 2.5) which was statistically significant, *p* = 0.01. There was a small but not statistically significant increase in the number of women referred in from other health centres, 54.6% (SD ± 0.7) vs. 55.1% (SD ± 1.6), *p* = 0.52.

### Maternal morbidity and mortality

**Fig. 2:**
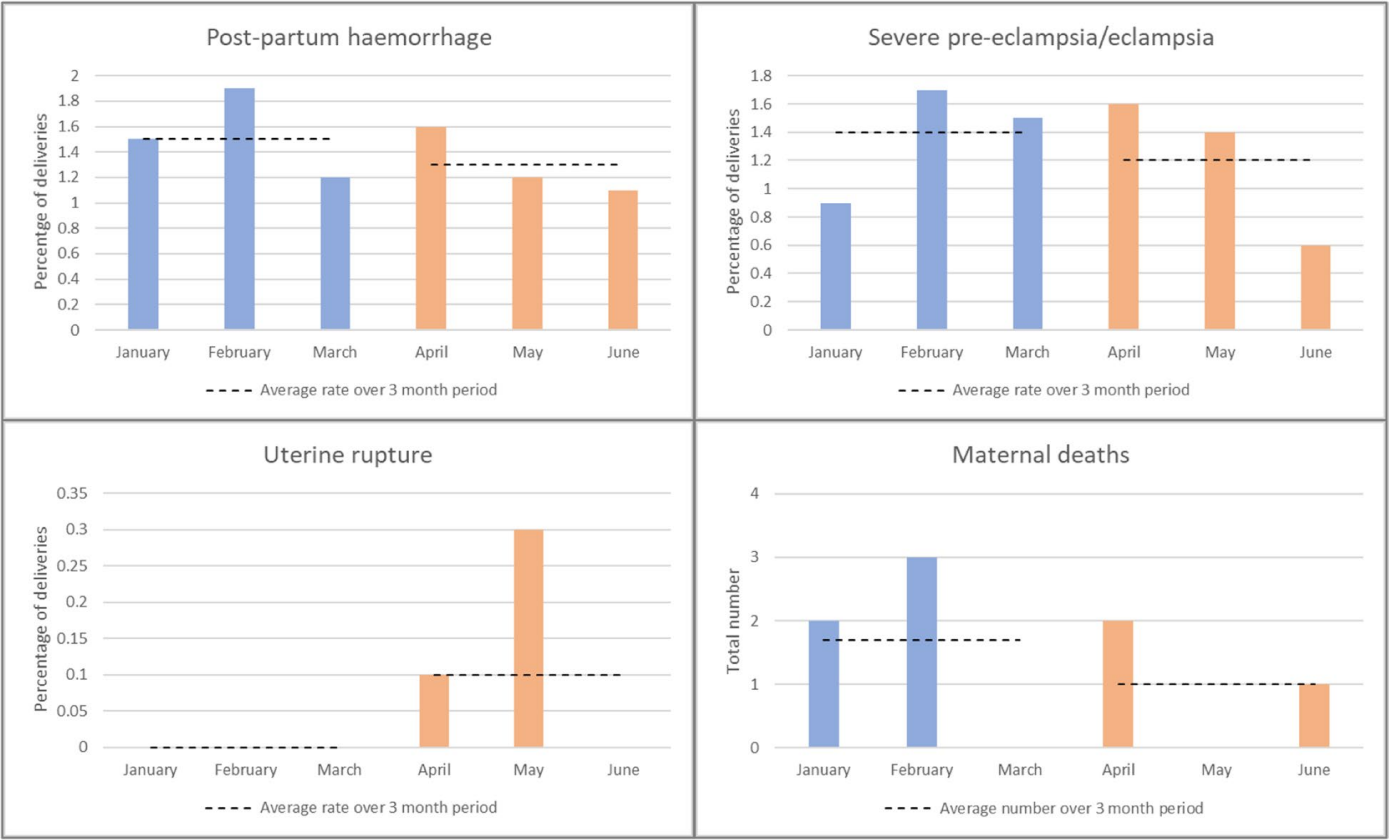
Maternal morbidity and mortality

Five maternal deaths were recorded in the period January-March 2020, and three maternal deaths were recorded in the period April-June 2020. There was no significant change in rates of PPH, 1.5% (SD ± 0.4) vs. 1.3% (SD ± 0.3), *p* = 0.75 or severe pre-eclampsia/eclampsia, 1.3% (SD ± 0.4 vs. 1.2% (SD ± 0.5), *p* = 0.74. There were more cases of uterine rupture reported in the second quarter of 2020, but the numbers remain very small, and there were not statistically significant, *p* = 0.39 (Fig. 2).

### Perinatal morbidity and mortality

**Fig. 3:**
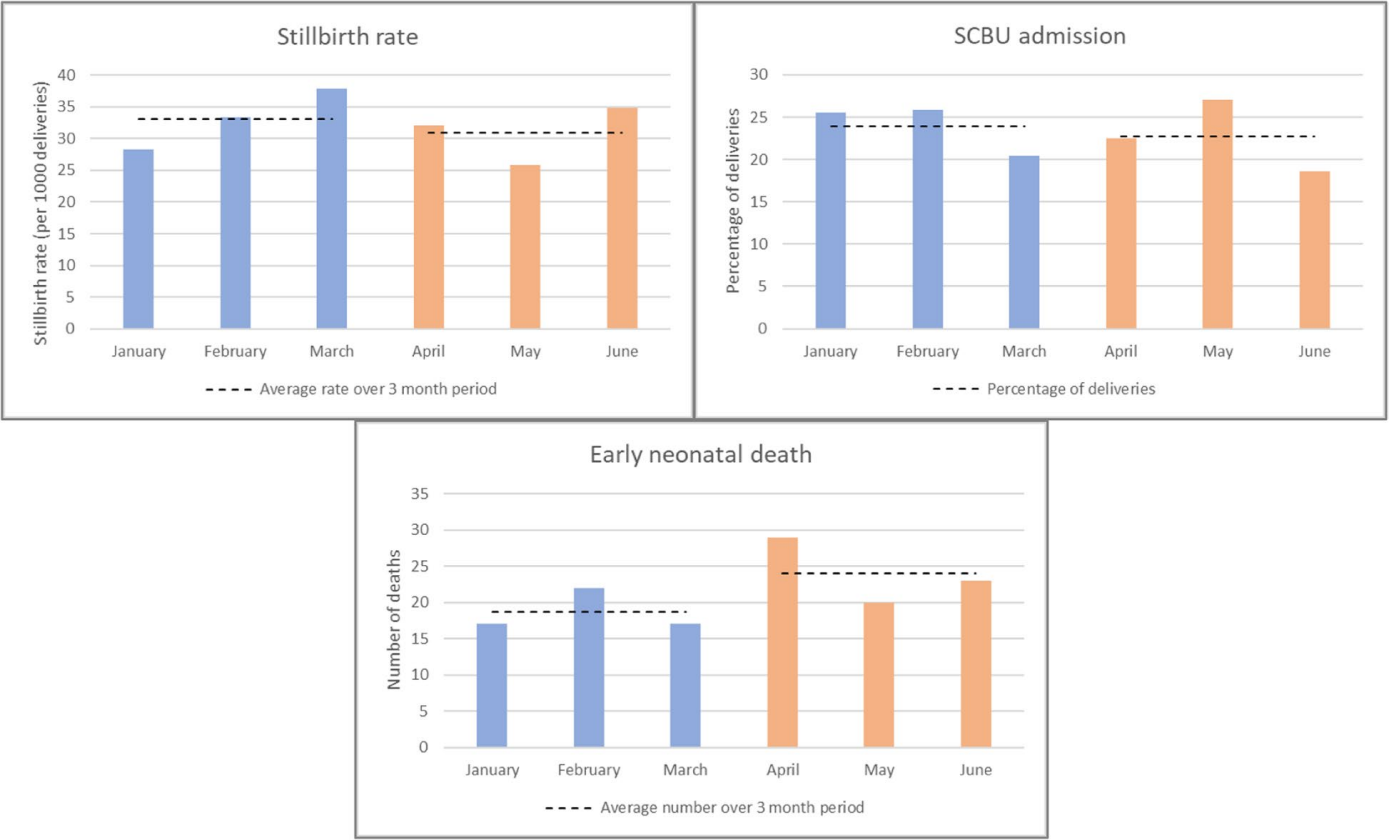
Perinatal morbidity and mortality

The stillbirth rate fell slightly from a mean of 33.1/1000 (SD ± 4.8) deliveries in the period January-March, to 30.9/1000 (SD ± 4.6) deliveries in the period April-June but this was not statistically significant, *p* = 0.81. The mean total number of early neonatal deaths (ENND) increased (mean 18.7 (SD ± 2.9) versus 24.0 (SD ± 4.6), but this was not statistically significant, *p* = 0.32, while the percentage of admissions to Special Care Baby Unit (SCBU) remained stable (mean 23.9 (SD ± 3.1) versus 22.7% (SD ± 4.3), which was not statistically significant *p* = 0.88 (Fig. 3).

## Discussion

A major strength of this study is that it relies on routinely collected and contemporaneously reported data from the maternity department. This means that data is readily and quickly available to monitor outcomes and trends, and any systematic errors in data collection are likely to remain constant from month to month. During the study period there was no statistical difference in the number of man-hours worked before and during the lockdown, indicating that the unit received the usual personnel coverage, hence there was little changes in clinical outcomes. However, it is possible that if wards were to be extremely short-staffed and staff working under additional pressure due to the pandemic, this may affect the way they report outcomes, for example, being less likely to recognise and report cases of post-partum haemorrhage.

The limitation is that the measures chosen may not perfectly represent the outcomes of interest. For example, antenatal care booking status represents coverage, but not quality of antenatal care provided. Additionally, none of the indicators selected represent important structural measures of quality. Statistical analysis was limited by small numbers of events, for example uterine rupture, and small sample sizes. Finally, our findings must be interpreted in the context of the limited pandemic impact at the time of the study. Maternal and perinatal outcomes may change as the pandemic progresses, and it will be important to monitor this.

The overall reduction in number of deliveries being conducted at Mpilo raises the question of where women are delivering instead. It would be interesting to examine statistics from other clinics within the city to see if their deliveries have increased. More women may choose to deliver locally, perceiving increased risks of Covid-19 at the central hospital or difficulties with transport. Some families moved back from urban to rural areas when the lockdown was announced and may be delivering at rural clinics and district general hospitals. The critical question is whether these women are still delivering in safe environments and complications recognised, and treated appropriately to prevent poor outcomes.

If fewer Caesarean sections were being done, this may be in part due to cancellation of elective Caesarean sections, but also reduced capacity in maternity theatres associated with limited resources. Emergency Caesarean sections are known to be associated with more complications for both the mother and the baby than elective procedures, however these outcomes are not captured by our data.

The number of women presenting in labour with an unbooked pregnancy had almost doubled since Covid-19 lockdown measures were instituted. The antenatal clinic at Mpilo has been closed during the lockdown period, and no women had been able to book their pregnancies. This trend therefore continued until antenatal services were restored. Women were probably booking elsewhere, for example at local clinics, which in time were reflected in an increase in referrals from other health centres. We would expect an increase in adverse outcomes to occur as fewer women access antenatal care. It could have been too soon, however, to observe the impact on women who would have booked their pregnancies during lockdown and would present for delivery later in the year.

Overall there had been no significant change in maternal outcomes. The small increase in cases of uterine rupture probably reflected barriers in accessing and delivering obstetric care on labour ward, however the numbers for all the outcomes are too small that it was difficult to draw conclusions. The increase in early neonatal deaths could have been due to problems within the obstetric or neonatal departments at Mpilo, at referring health centres, or delayed presentation associated issues within the community such as transport. Further investigation would be needed to clarify these issues.

Finally, the data on maternal deaths recorded here does not capture deaths occurring outside hospital or at other health centres and hospitals within the region. It would be important to explore this data for the whole region when it is available, to see whether women are experiencing excess morbidity and mortality outside Mpilo, unable to access the central hospital itself especially during future lockdown periods.

Similarly to many southern African countries, the healthcare system in Zimbabwe is fragile and vulnerable to the effects of internal and external crises such as Covid-19 [19]. In Zimbabwe, the Covid-19 pandemic has occurred on the background of a weakened healthcare system that has experienced limited resources. Our study shows that it is possible to provide enough man-hours during lockdown periods and prevent adverse outcomes in contrast to our hypothesis that maternal and perinatal morbidity and mortality would increase during the lockdown period. Interestingly, a study of the effects of Covid-19 lockdown measures on access to primary care in rural South Africa had similar unexpected results [20].

The results presented here could also be seen to represent the significant efforts of healthcare workers and maternity services showing remarkable resilience in difficult circumstances, with great commitment to keeping women and their babies safe. Several elements of organisational design, commonly used to analyse strategic success [21], may describe the way that resilience is manifested at Mpilo hospital to prevent adverse outcomes. The structure and systems of the Mpilo maternity department are oriented to identifying women at risk of complications and intervening early. The focus is on reducing morbidity and preventing mortality by early recognition and timely involvement of senior midwifery and medical staff. Regular rounds by maternity matrons identify issues and escalate promptly to consultants if necessary. Both medical and midwifery staff are resilient and adaptable, with experience of working in difficult conditions. They are skilled at responding to complex emergencies and scenarios on labour ward such as eclampsia, uterine rupture and major PPH, with competencies such as Caesarean hysterectomy as routine. They are conditioned to working with limited availability of resources. Finally, leadership style in the department tends to be formal and directive, with an emphasis on accountability. This may not suit all environments but can provide clear and consistent guidance when trying to achieve specific goals and maintain standards in challenging circumstances such as those presented by Covid-19.

While the results presented here are positive, it will be essential to continue monitoring outcomes as the pandemic in Zimbabwe escalates and further lockdown measures and changes to services are being made. There is still potential for poor maternal and perinatal outcomes if access to healthcare remains restricted and healthcare workers are increasingly burnt out by the worsening pandemic.

## Conclusions

Healthcare systems in southern Africa are vulnerable to the indirect effects of Covid-19. There is potential for lockdown measures to have adverse effects on maternal and perinatal outcomes in Zimbabwe and it will be important to continue monitoring these as the pandemic unfolds. It is critical to maintain or improve the man-hours where possible to continue giving reasonable coverage in the unit. Overall, maternity services at Mpilo showed resilience during the lockdown from April-June 2020, with no significant increase in major adverse outcomes. Further studies would be beneficial to explore women’s experiences and understand how bookings and deliveries at local antenatal clinics and health centres changed during this time when fewer accessed the central hospital. Lessons may be learnt that could improve preparedness for similar pandemic events in future.

## Data Availability

The datasets used during the current study are available from the corresponding author on reasonable request.

## Abbreviations

ANC: Antenatal care
ENND: Early neonatal death
LMIC: Low and middle income country
PPH: Postpartum haemorrhage
SCBU: Special care baby unit
SD: Standard deviation
WHO: World Health Organisation
